# Evaluation of a School-Based Depression Prevention Program among Adolescents from Low-Income Areas: A Randomized Controlled Effectiveness Trial 

**DOI:** 10.3390/ijerph110505273

**Published:** 2014-05-15

**Authors:** Karlijn C. M. Kindt, Marloes Kleinjan, Jan M. A. M. Janssens, Ron H. J. Scholte

**Affiliations:** Behavioural Science Institute, Radboud University Nijmegen, P.O. Box 9104, 6500 HE Nijmegen, The Netherlands; E-Mails: m.kleinjan@bsi.ru.nl (M.K.); j.janssens@bsi.ru.nl (J.M.A.M.J.); r.scholte@bsi.ru.nl (R.H.J.S.)

**Keywords:** depression prevention, adolescents, selective prevention, low-income, school based, RCT, parental psychiatry, minority youth, Op Volle Kracht

## Abstract

A randomized controlled trial was conducted among a potential high-risk group of 1,343 adolescents from low-income areas in The Netherlands to test the effectiveness of the depression prevention program Op Volle Kracht (OVK) as provided by teachers in a school setting. The results showed no main effect of the program on depressive symptoms at one-year follow-up. A moderation effect was found for parental psychopathology; adolescents who had parents with psychopathology and received the OVK program had less depressive symptoms compared to adolescents with parents with psychopathology in the control condition. No moderating effects on depressive symptoms were found for gender, ethnical background, and level of baseline depressive symptoms. An iatrogenic effect of the intervention was found on the secondary outcome of clinical depressive symptoms. Based on the low level of reported depressive symptoms at baseline, it seems that our sample might not meet the characteristics of a high-risk selective group for depressive symptoms. Therefore, no firm conclusions can be drawn about the selective potential of the OVK depression prevention program. In its current form, the OVK program should not be implemented on a large scale in the natural setting for non-high-risk adolescents. Future research should focus on high-risk participants, such as children of parents with psychopathology.

## 1. Introduction

Depression rates are known to rise in adolescence. Among 13- to 17-year olds, almost 17% of girls and almost 9% of boys reported a lifetime prevalence of a major depressive episode [1]. A meta-analysis of more than 61,000 children and adolescents revealed that girls reported a significant increase in depressive symptoms from the age of thirteen [2]. Boys reported relatively stable levels of depressive symptoms across adolescence. To attenuate the increase of depressive symptoms and reduce the incidence of depressive disorders, several group depression-prevention programs have been developed in the last two decades (e.g., Penn Resiliency Program [3], LISA-T [4], Coping with Stress Course [5]). The reviews of depression prevention programs reported lower depressive symptoms in adolescents who received a prevention program compared to a control group that did not receive the intervention. However, studies reported inconsistent findings [6,7,8]; therefore, additional research is needed to determine what caused these inconsistencies. Previous studies have indicated that it would be necessary to test whether adolescents with risk factors to develop depressive symptoms benefit from depression prevention programs [8,9] and whether these programs are effective when implemented in natural settings (*i.e.*, incorporated into schools) [6,8]. The aim of the current study is to test the effectiveness of a depression prevention program on a selective group of adolescents from low-income areas when implemented in a school setting under real-life conditions. 

Generally, three levels of prevention are distinguished: (1) universal prevention targeting entire populations, (2) selective prevention targeting subgroups with an increased risk to develop symptoms of a given disorder, and (3) indicated prevention targeting individuals with elevated symptoms of a given disorder who do not meet the criteria of the disorder [9]. The latter two levels are also described as targeted prevention. In a recent meta-analytic review, evidence was found that both universal and targeted depression prevention programs are effective in preventing depressive symptoms compared to no intervention [10]. However, earlier reviews found that targeted depression prevention with adolescents have larger effect sizes in preventing depressive symptoms compared to universal approaches [7,8]. There are several reasons why a targeted approach, compared to a universal approach, will lead to larger effect sizes [9]. First, in a selective approach, participants are more likely to increase in depressive symptoms over time, making it easier to detect a preventive effect of the intervention in the experimental condition compared to the control condition. Furthermore, a decrease of symptoms can only be found within those adolescent who have elevated levels at baseline, and thus, the effect size is more likely to be larger in the selective group. A meta-analysis of 17 PRP-studies with almost 2,500 young adolescents showed that adolescents who received the program reported fewer depressive symptoms directly after the intervention and at 6 and 12 months follow-up compared to a non-intervention control group [6]. Moreover, universal, selective, and indicated prevention studies on PRP showed a significant effect on depressive symptoms, although the targeted prevention tended to be more effective compared to a universal approach [6]. Recently, a large universal study implemented and tested an adapted version of the PRP in the school curriculum in the UK [11]. The study found lower levels of depressive symptoms at post intervention, although the effect was small, and it did not persist at 1-year and 2-year follow-up. 

The research in The Netherlands that tested a translated and adapted version of the Penn Resiliency Program (PRP) [3], which was named Op Volle Kracht (OVK) [12], showed strong effects on the study sample [13], but in contrast to the PRP studies in the U.S., no effects were found in a universal sample [14]. Because previous studies have found indicated but no universal prevention effects of OVK, the current study will test whether OVK has a selective prevention effect on targeted adolescents that live in lower income areas in The Netherlands. These adolescents may be more vulnerable to depressive symptoms because they are exposed to poverty and negative life events [15,16,17]. Therefore, we selected only schools with a high proportion of adolescents from low-income areas and administered the program to entire classes. Such a selective approach has advantages for practice compared to indicated and universal prevention. First, individuals are not separated from their group during a prevention program and thus no stigmatization occurs [18]. Second, the costs of screening are avoided, since the program could be delivered to already existing groups, which are known to have a higher risk for developing depressive symptoms. Lastly, a larger group of vulnerable adolescents will be reached, including the adolescents who may not be at an immediate risk but who may be at risk at a longer term.

When depression prevention programs are effective in a research setting, is it important to test whether these programs are as effective when they are implemented in a natural setting. Previous research has found PRP to be effective under research conditions that are difficult to implement on a larger national scale. That is, the adolescents received the program in small groups with a maximum of 14 participants and received the training after school time what caused a low attendance rate, and moreover, the teachers were intensively supervised and checked on integrity [3,19,20]. However, with this approach, PRP had significant effects when provided by teachers [6]. Thus, it is important to test the effectiveness of the program when it is fully incorporated into schools, because that will provide additional knowledge whether the program also has a beneficial effect on depression prevention when it is implemented on a large scale. Moreover, concerning the feasibility of a large-scale implementation, it is necessary that the costs of mental health care are manageable. A school-based approach, including implementation by teachers in a classroom setting, may serve this purpose. In the current randomized controlled selective prevention study, we examined whether the depression-prevention program OVK is effective for 11 to 16 year old adolescents from low-income areas in the Netherlands when implemented at a class level as a part of the school curriculum and facilitated by trained teachers. We hypothesized that the OVK group would experience milder increase in depressive symptoms compared to the control group at one-year follow-up.

Additionally, previous research has demonstrated the importance of establishing subgroups that are more likely to derive benefit from a depression prevention program [8,9]. Based on theory and previous research, we will explore whether gender, clinical depressive symptoms at baseline, ethnical background, and parental psychopathology moderate the intervention effects.

### 1.1. Gender

Boys and girls may respond differently to depression prevention programs [21], as some studies showed that girls benefitted more from these programs compared to boys [8,20,22,23]. Other studies showed that boys improved more [24] while still others did not find such differences [25]. 

### 1.2. Baseline Depressive Symptoms

Previous studies have found larger effects for groups with elevated baseline depressive symptoms compared to low symptom groups [19,20,25]. However, it is important to know whether participants with elevated depressive symptoms can also benefit from a class based group program in which all adolescents in a classroom receive the program irrespective of their level of depressive symptoms. We hypothesized that adolescents with elevated depressive symptoms will profit more from the program than their peers with lower depressive symptoms.

### 1.3. Ethnical Background

Depression prevention programs are developed and tested primarily in the United Stated and Europe [8,10]. Consequently, these programs may be less effective for ethnic minority groups because of cultural differences in dealing with depressive symptoms, or the programs may not adequately address the life circumstances that minority youth face [8]. On the contrary, a larger effect may be expected for minority groups, because this group reports more internalizing problems and depressive symptoms [26,27]. The PRP program has been found to be effective for Latino but not African American youth [17], yet, more research is necessary to determine whether ethnic background moderates the effects of depression prevention programs. In the current study, we examined the moderating effects of ethnic background by comparing the effect of the OVK program on native adolescents and ethnic minority youth.

### 1.4. Parental Psychopathology

Adolescents of parents with psychopathology have an increased risk for developing a depressive disorder [28] with an earlier onset and a more malignant course [29]. We hypothesized that the adolescents with parents with psychopathology would profit more from the OVK intervention. 

### 1.5. Motivation and Perceived Atmosphere.

In addition, theoretical models like the Theory of Planned Behavior [30] and the Transtheoretical model [31] imply that individual’s readiness to try and change is an immediate precursor of behavior change. Among the intervention group, the effect of the program might thus be influenced by the motivation of participants to attend the program and to try to internalize the offered skills. Moreover, a negative atmosphere in groups might influence the effects of an intervention. A negative atmosphere could arise due to tediousness or adolescents’ behavioral problems [20]. Although externalizing problems have not been found to moderate the effect of a depression prevention program [22], it is unclear whether the perceived atmosphere can differentiate within the experimental group for whom the program works. For these reasons, we assessed the motivation before the start and during OVK, as well as the perceived atmosphere during the OVK lessons in those adolescents who were assigned to the intervention condition. 

## 2. Method

### 2.1. Design and Randomization

A non-blind two-arm parallel group clustered randomized controlled trial was used to test the effectiveness of the OVK program among adolescents from low-income areas. Randomization was conducted within schools at the class level to control for school characteristics, with allocation ratio of 1:1. The sample was stratified by level of education (high *versus* low). An independent researcher from the research institute used a computerized random number generator with a blocked randomization scheme (block size 2) to perform the allocation. This resulted in a list of classes that were allocated to control or intervention condition, which was communicated to the school by the first author.

### 2.2. Participants and Procedure

Eligible participants for the study were adolescents in the 7th and 8th grades (age 11–16 years, M = 13.42, SD = 0.77) attending secondary schools in the Netherlands that met the criterion that at least 30% of their pupils lived in low-income areas. A list of these schools was obtained from the Dutch Ministry of Education, Culture and Science based on the average household income in the postal code area where the adolescent lived. Schools received a written invitation to join the study. The principals were asked to allow the teachers of the experimental group to complete the training in the OVK program and implement the OVK lessons within the curriculum. After two weeks, the schools were contacted by telephone. If they agreed to participate, a member of the research team visited them and selected classes. The parents received a letter about the content and the aim of the program, describing the study procedures. Parents were asked to provide permission for their child’s participation through passive consent. The data were not collected from adolescents whose parents did not give permission. Yet, their attendance in the program was obligatory, since it was included in the regular school curriculum. The teachers of the classes that were assigned to the OVK condition were invited to a four-day training conducted by the research team. The participants completed online self-report questionnaires during school hours at baseline (T1, December 2011), post intervention (T2, June 2012), and at six and twelve months follow-up (T3, December 2012 and T4, June 2013). It took approximately 50 min to complete the questionnaire. Teachers supervised the measurements following strict guidelines when assigning personal codes to adolescents and assuring their privacy during administration. Only at T4, two schools asked for support and members of the research team assisted with paper questionnaires. Incentives were given at T3 and T4 to enhance response rates. At T3, the teachers of the school were offered a cream cake when they reached a response of 90% or more. At T4, the schools received 10 euro’s per completed questionnaire, which could be spent either for the school or could be given to the individual pupils. We registered this trial in the Netherlands Trial Register (No. NTR3110), and the local Ethical Committee (ECG13042011) approved the study protocol, which was published [32]. 

### 2.3. Intervention

Participants assigned to the experimental condition received 16 weekly lessons of OVK during school hours by their mentor, who is the designated teacher to whom parents and adolescents first can turn to in case of problems. In OVK, the adolescents are taught skills derived from cognitive behavior therapy (CBT), and social problem-solving and coping skills, through completing pen and paper exercises in a workbook, engaging in the group discussions, performing role-plays, and completing homework assignments. First, they learn about associations among situations, cognitions, feelings, and behavior. Then they learn to check the accuracy of their cognitions and to be flexible in finding alternative interpretations. Further, they learn social and coping skills, including negotiating, assertiveness, and relaxation. The content of the OVK program is described in detail elsewhere [12]. The teachers were extensively trained in four days by certified members of the research team. The first two days consisted of “adult skills”, in which the teachers were taught to use the CBT skills on their own thoughts and feelings. During the third and fourth day the teachers practiced all the lessons of the program, on which the trainers provided feedback. Moreover, the teachers were given a comprehensive manual that thoroughly described each exercise. In addition, we gave precise instructions as to what components might be cut in case of lack of time, as recommended by Ringwalt [33]. During the program, the teachers could contact the research team at any time in case they had questions about the content. They were asked to report how many lessons they had taught. Of the 28 teachers who provided the OVK lessons, 16 filled out adherence reports. On average 80.5% of 16 lessons were taught per class, with 95.3% of the first eight lessons and 65.5% of the last eight lessons being taught. After the OVK program ended, we interviewed all teachers for a qualitative process evaluation.

### 2.4. Measurements

#### 2.4.1. Depressive Symptoms

The primary outcome, *i.e.*, depressive symptoms, was assessed using the Children’s Depression Inventory (CDI) [34]. The CDI is a self-report questionnaire comprising 27 items assessing affective, cognitive, and behavioral symptoms of depression. The item on suicide was removed in the current study to optimize collaboration with school officials and parents [14]. For each item, participants selected one of the three statements that best applied to them during the last two weeks measured on a 3-point scale from zero to two (e.g., “I feel like crying once in a while” (0), “I feel like crying on most days” (1), “I feel like crying every day”(2)). The CDI has shown good internal consistency and validity [35]. Cronbach’s alpha for the CDI in the current study ranged from 0.85 to 0.90, indicating good internal consistency. The sum of depressive symptoms was calculated by multiplying the mean score with 27 to facilitate the comparability with other studies [36,37]. When comparing our study with those studies that did include all items, it is important to keep in mind that the allocation to the subgroup clinical depressive adolescents might have been slightly different.

#### 2.4.2. Clinical Depressive Symptoms

Adolescents who had a CDI-sum score of 19 or higher were labeled as having clinical depressive symptoms, as is the recommended cut-off score used in population samples [38,39].

#### 2.4.3. Ethnical Background

Adolescents were asked in which country they and their parents were born. When the adolescent or one of the parents was not born in The Netherlands, the adolescent was classified as ethnic minority.

#### 2.4.4. Parental Psychopathology

At baseline, the adolescent was asked whether one of the parents had been treated by a psychiatrist. If their answer was “yes”, they were analyzed as youth with a parent with psychopathology.

#### 2.4.5. Motivation and Perceived Atmosphere

We measured motivation to attend the OVK-program at baseline, and the motivation and perceived atmosphere during the program at the post intervention assessments. Adolescents were asked “How motivated are you to start with OVK”/“How motivated were you during the OVK lessons” on a 4-point scale, ranging from “not motivated at all” to “very much motivated”. They responded to the question about perceived atmosphere in the class during the OVK program on a 7-point Likert scale ranging from “not good” to “good”.

### 2.5. Power Calculation

We conducted a power calculation, assuming a small effect size of Cohen’s d = 0.20 at 12 month follow-up [6]. We considered potential loss of power due to clustering of data in classes, a maximum of 20% attrition over time, and multiple imputations. The sample size per condition was fixed at 662 students (alpha < 0.05, power = 0.80).

### 2.6. Attrition Analyses

Attrition at T1 through T4 was analyzed with logistic regression analyses in which dropout was the dependent variable, and baseline depressive symptoms, demographics, and outcome variables were predictors. Adolescents lost to follow-up were more likely to be boys (*OR* = 1.69, CI 95% = 1.29–2.22, *p* < 0.05), ethnic minority youth (*OR* = 0.614, CI 95% = 0.464–0.814, *p* < 0.05), and older pupils (*OR* = 0.915, CI 95% = 0.711–1.177, *p* < 0.05), and they were more likely to have higher depressive symptoms at baseline (*OR* = 0.375, CI 95% = 0.219–0.641, *p* < 0.05).

### 2.7. Strategy of Analyses

Baseline differences between conditions were tested with independent *t*-tests and Chi square analyses to assess whether randomization resulted in a balanced distribution of demographic and outcome variables. 

The intervention effect on depressive symptoms at one year follow-up (T4) was analyzed by means of multivariate regression and logistic regression analyses in Mplus 7 [40] while controlling for clustering of data in classes. The dependent variables in the analyses were the continuous primary outcome variable of depressive symptoms and the dichotomous secondary outcome variable of clinical depressive symptoms. Baseline depressive symptoms and the variables for which we found baseline differences across conditions were included as covariates. Subsequently, moderating effects of gender, clinical depressive symptoms at baseline, ethnical background, and parental psychopathology were tested by computing the interaction effects of these variables with condition.

The data was analyzed by means of completers only (N = 948) and intention to treat (N = 1,324). In the intention to treat approach, missing values on the primary outcome variable, that is, depressive symptoms were imputed for all four measurements using 20 imputation sets by multiple imputations in SPSS 19. Imputations were done separately for the control group and intervention group, and variables that correlated significantly with depressive symptoms were used as auxiliary variables [41]. In addition, we performed sensitivity analyses for the secondary outcome variable of clinical depression with assumptions of extreme high or low values for missing data to assess the influence of loss to follow-up and to test the robustness of the results [42]. 

Post-hoc analyses were conducted to examine the treatment and prevention effects of OVK on linear increase or decrease of depressive symptoms by means of a latent growth curve approach. A treatment effect is characterized by a stronger decrease of symptoms in the intervention group compared to the control group after the intervention has taken place. A prevention effect is characterized by an increase of symptoms in the control group and a smaller increase or no increase of symptoms in the intervention group during the course of the program or during the months or years after the intervention [7]. Applying a latent growth curve approach allows us to estimate the individual growth curve of depression for each subject, including an initial level (intercept), and a certain rate of change over time (slope). A latent growth curve model can use repeated measures of depression over time as indicators of latent variables that describe group mean trajectories while allowing for between-individual differences in trajectories [43]. In this linear growth model, the depressive symptoms slope was regressed on the intervention condition variable. It controlled for clustering effects, as adolescents were nested within classes. We controlled for the variables for which we found baseline differences.

For the OVK-group, we conducted independent t-tests to analyze differences in depressive symptoms at T4 between high and low motivated adolescents and between adolescents who rated the atmosphere during the program as good/neutral and bad. The effect of motivation and atmosphere on depressive symptoms at one year follow-up (T4) was analyzed by means of multivariate regression [40] in which we controlled for clustering of data in classes and baseline depressive symptoms.

## 3. Results and Discussion

**Figure 1:**
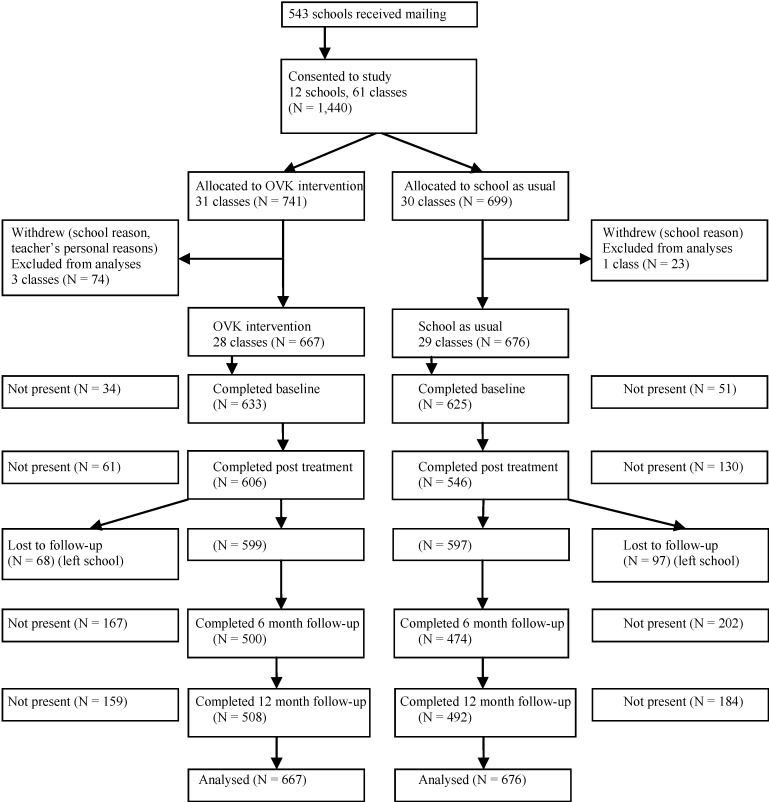
Flow diagram.

Note: OVK = Op Volle Kracht.

In total, 543 schools received a written invitation to participate in the study. In the summer of 2011, 12 schools agreed to participate, providing access to 1,440 adolescents from 61 classes in which the randomization was conducted before baseline assessment. Before the start of the intervention, four classes dropped out: one school with two classes withdrew and two teachers in the intervention condition could not participate for personal reasons. Hence, these classes were considered as dropouts and excluded from the study. Finally, 1,343 adolescents from 57 classes in 11 schools participated in the study, and they were analyzed for the primary outcome. In total, 667 adolescents were allocated to the OVK depression prevention program and 676 adolescents to the control condition receiving the regular school curriculum. The participation rates at baseline and post intervention measurements were good (T1: 93.7% and T2: 85.8%). After T2, 12.3% dropped out due to change of schools and by consequence, the participation rates decreased at two follow-up assessments (T3: 72.5% and T4: 74.5%). See Figure 1 for the participant flow diagram.

### 3.1. Descriptive Statistics

The baseline characteristics, mean depressive symptoms, and percentages of clinical depressive symptoms for all measurements are shown in Table 1 and Table 2. Descriptive analyses showed significant differences between the conditions in baseline parental psychopathology (*X^2^* [1] = 3.87, *p* = 0.049). The control condition included a greater number of adolescents who had parents with psychopathology. Therefore we used parental psychopathology as a covariate in all analyses.

**Table 1:** Baseline characteristics.

Characteristic	OVK Condition N = 667	Control Condition N = 676	Total N = 1,343	Significant Difference I-C
	*M* (*SD*)	*M* (*SD*)	*M* (*SD*)	
Gender (%)				ns.
Girls	51.1	53.4	52.3	
Boys	48.9	46.6	47.7	
Age	13.42 (0.79)	13.42 (0.76)	13.42 (0.77)	ns.
Ethnical background (%)				ns.
Native Dutch	46.0	49.4	47.7	
Ethnic minority	54.0	50.6	52.3	
Parental psychopathology (%)	4.6	7.2	5.9	*p* < 0.05
School level (%)				ns.
Low	46.8	44.5	45.6	
High	53.2	55.5	54.4	
Sum depressive symptoms	8.56 (6.68)	8.55 (6.33)	8.55 (6.51)	ns.
Clinical depressive symptoms (%)	7.4	6.9	7.2	ns.

Notes: Logistic regression analyses were used to calculate differences between Intervention (I) and Control (C); ns. = non significant.

**Table 2:** Means (and standard deviations) of sum depressive symptoms and percentages of clinical depressive symptoms at all measurements (T1–T4) separately for conditions.

Outcomes	T1	T2	T3	T4
Sum depressive symptoms	8.55 (6.51)	9.47 (7.72)	9.64 (8.19)	9.62 (8.48)
OVK	8.56 (6.68)	9.53 (7.98)	9.17 (7.99)	10.00 (9.14)
Control	8.55 (6.32)	9.39 (7.43)	10.14 (8.37)	9.22 (7.74)
Clinical depressive symptoms (%)	7.2	9.8	12.1	13.2
OVK (%)	7.4	10.5	10.4	16.3
Control (%)	6.9	9.0	13.8	10.0

### 3.2. Main Effects of OVK on Depressive Symptoms

Regression analyses did not reveal the main effects at 12 months follow-up for the OVK intervention on the primary outcome of depressive symptoms, both for Completers Only (CO) (*β* = −0.049, SE = 0.041, *p* = 0.24) and Intention To Treat (ITT) (*β* = −0.044, SE = 0.037, *p* = 0.23). An iatrogenic main effect of the OVK intervention was found in CO and ITT analyses on the secondary outcome of clinical depressive symptoms (CO: *OR* = 0.558, 95% CI = 0.351–0.887, *p* < 0.05; ITT: *OR* = 0.564, 95% CI = 0.378–0.842, *p* < 0.01). Compared to the control group, more adolescents in the OVK group reported depressive symptoms above the clinical cut off at 12-months follow-up.

#### Sensitivity Analysis

Because the power for the current study was calculated based on our primary outcome of depressive symptoms and not on the secondary dichotomous outcome of clinical depressive symptoms, we wanted to check the robustness of the iatrogenic effect on clinical depressive symptoms with a sensitivity analysis. We did not conduct a sensitivity analysis for the primary outcome of depressive symptoms because (a) the power was calculated to detect significant effects on this outcome and (b) the regression analyses on the primary outcome convincingly showed no significant main effect. Sensitivity analyses for the primary outcome were not expected to lead to a different conclusion. 

We conducted a sensitivity analysis for the secondary outcome of clinical depressive symptoms with two scenarios. In the first scenario, we imputed non-clinical depressive scores (*i.e.*, best case scenario) for all missing data at T4. In the second scenario, we assumed clinical depressive scores (worst case scenario). Regardless the scenario we used, the absolute difference of the amount of adolescents with clinical depressive symptoms between the control condition and the OVK condition is similar. However, the relative difference will be higher in the best case scenario compared to the worst case scenario, and will thus easier result in a significant result. In the conducted sensitivity analysis, the iatrogenic effect was found only in the best-case scenario (OR = 0.576, CI 95% = 0.366–0.908, *p* ≤ 0.05) but not in the worst-case scenario (*OR* = 0.893, CI 95% = 0.578–1.380, *p* = 0.61). Thus, this indicates that the iatrogenic effect might be influenced by the manner in which missing data is dealt with.

### 3.3. Moderation

**Figure 2:**
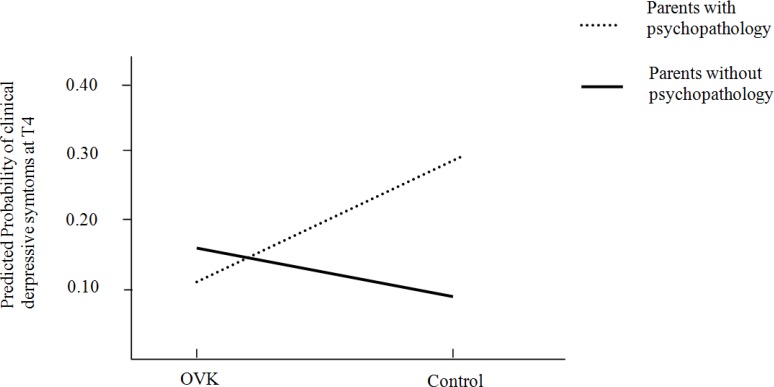
Interaction effect for adolescents with/without parents with psychopathology and the intervention.

No moderation effect on the relation between condition and depressive symptoms was found at T4 for gender, clinical depressive symptoms at baseline, ethnic background, and parental psychopathology for both ITT and CO. Regarding the relation between condition and clinical depressive symptoms, only parental psychopathology was found to have moderating effects on the outcome for the completers only group (CO: *OR* = 0.121, CI 95% = 0.029–0.505, *p* < 0.01) and a marginal effect for the intention to treat group (ITT: OR = 0.239, CI 95% = −0.155–0.633, *p* = 0.087). Concerning the prevention of clinical depressive symptoms, adolescents who received the OVK-program and had parents with psychopathology showed significantly lower levels of depressive symptoms compared to adolescents who were in the control condition and had parents with psychopathology. More specific, the adolescents with parents with psychopathology had a probability of 0.11 to have clinical depressive symptoms at T4 when they received the program, to a probability of 0.29 for those who had parents with psychopathology and did not receive the program (see Figure 2).

### 3.4. Post-hoc Analyses

#### 3.4.1. Latent Growth Curves

*Post-hoc* analyses were conducted to examine the effect of the OVK program on linear increase or decrease in depressive symptoms by means of latent growth curve approach. First, we tested the initial developmental model (*i.e.*, no predictors) for depressive symptoms. The fit between the data and the model was excellent (*X^2^* (df = 5)) = 12.804, *p* < 0.05, RMSEA = 0.032, CFI = 0.981, TLI = 0.977). The intercept was significant (*β0* = 1.17, *p* < 0.001) but the slope was not (*β1* = 0.014, *p* = 0.82), indicating that participants scored above zero on depressive symptoms at baseline and that depressive symptoms remained stable over time. The variance of the slope was significant (*p* < 0.001). We additionally tested for possible quadratic trends, but these were not significant. Study condition was included as a predictor of depressive symptoms, and we controlled for parental psychopathology to test the effect of the intervention on the slope. Condition was unrelated to the intercept (*β* = −0.012, *p* = 0.77) or the slope of depressive symptoms (*β* = −0.021, *p* = 0.73), indicating that the increase or decrease in depressive symptoms across follow-ups did not differ between the intervention and control condition. The fit between the model and data was excellent (*X^2^* (df = 9) = 24.48, *p* < 0.01, RMSEA = 0.035, CFI = 0.976, TLI = 0.963).

#### 3.4.2. Motivation

Adolescents who attended the OVK program reported various levels of motivation before and during the course of the OVK lessons: not motivated at all (11.4% before, 22.9% during), a little bit motivated (22.9% before, 41.9% during), pretty much motivated (29% before, 22.7% during), very much motivated (6.0% before, 6.0% during), and did not know (24% before, 6.4% during). Most participants rated the atmosphere during the OVK lessons as neutral (40.0%) or positive (38.7%). An independent *t*-test revealed no difference between high and low motivated adolescents, as reported *before* the intervention started, in depressive symptoms at T4 (*t* = 1.66, *p* = 0.099). However, significantly lower depressive symptoms were found at T4 for adolescents who reported to be motivated during the program compared to low-motivated adolescents (*t* = 2.57, *p* < 0.05). Additionally, adolescents who rated the atmosphere during the program as neutral to good reported less depressive symptoms at T4 compared to those who rated the atmosphere as bad (*t* = 3.15, *p* < 0.01). Additional regression analyses, in which we controlled for depressive symptoms at T1, revealed that motivation *during* the program (*β* = −0.124, SE = 0.056, *p* < 0.05) and the reported atmosphere (*β* = −0.102, SE = 0.049, *p* < 0.05) predicted depressive symptoms at T4. The participants who rated the atmosphere in the class during the OVK program as more positive and had higher motivation had less depressive symptoms at T4. 

### 3.5. Main Findings

The present study examined the effectiveness of the depression prevention program OVK by means of an RCT-design in a selective population of 11 to 16 year old adolescents from low-income areas in The Netherlands. There are three important findings that will be discussed in detail below. First, contrary to what we expected, we did not find an effect of the OVK program on the primary outcome of depressive symptoms one year after the intervention. Second, we found an interaction effect of condition with the presence of parental psychopathology. Third, we found an iatrogenic effect on clinical depressive symptoms at one-year follow-up. In contrast to other studies, we did not find any preventive effects on depressive symptoms, what may be explained by factors that have previously been identified as predictive of the impact of depression prevention programs, namely participants, providers, content, and study design [8].

#### 3.5.1. Participants

First, our participants had baseline depressive symptoms (*M* = 8.55) below the 70th percentile of depressive symptoms [36], which is only slightly higher than in universal depression prevention studies that were conducted in a non-high-risk, population sample (*M* = 8.29, [11]; *M* = 7.56, [14]). In a truly selective sample, adolescents would be expected to have higher baseline depressive symptoms and consequently have more room for improvement, which would increase the possibility to find significant effects [9]. Indeed, studies that found effects generally included participants who scored higher on depressive symptoms at baseline (e.g., CDI > 11 [44]; CDI > 12 [20] and CDI > 20 [13]). Additionally, the latent growth curves showed no changes in the development of depression over time in both the intervention and control groups, underlying the notion that our included adolescents are not at a risk to develop symptoms. Although adolescents from low-income are more vulnerable to develop depressive symptoms [15,16,17], this might not be true in The Netherlands because it is a high-income country with relatively high standards even in low-income areas. Moreover, in our selective group, at least 30% of the adolescents came from low-income areas, which means we cannot rule out that a significant proportion of the sample may not belong to a low-income group. It is, thus, debatable whether we actually reached a selective high-risk population. The program was not effective with the selective approach that we took. However taking a different selective approach might lead to better effects (e.g., selecting adolescents whose parents have a psychopathological disorder). In addition, we did not find a moderating effect for initial depressive symptoms, what suggests that the outcome did not differ for adolescents with elevated depression scores. Still, the program might be effective when selecting a homogeneous group of adolescents with elevated depression scores. In this latter case, discussed topics within the OVK program will probably be more recognizable for these adolescents, which could positively influence the interactions in the group and make the program more helpful. Another explanation why we did not find an intervention effect is that the participants might have been too young. The increase in depressive symptoms is found to be strongest for adolescents between 15 and 18 years old [45] while in our study, the mean age at baseline was 13 years. When delivering the program to older adolescents, the control group might experience an increase in depressive symptoms and a prevention effect might become apparent, which would be in line with reviews showing that depression prevention programs are more effective for older adolescents [6]. Because of their young age and the apparent absence of depressive symptoms, it could also be argued whether these young adolescents recognize themselves in the program’s used examples, what could make our sample less susceptible for the taught skills. This is in line with other studies on large scale, school-based CBT interventions with a similar age group that also did not find effects on depressive symptoms [46,47].

In the intervention group, we found that the program decreased the levels of depressive symptoms among adolescents reporting to be more motivated *during* the program. It might be that the motivated adolescents learned and used more skills from the program, as would be in line with earlier findings, which showed that motivation to learn is significantly related to course outcomes [48]. Moreover, the reported motivation *prior* to the training did not predict for whom the OVK training was effective, and thus it does not seem useful for further research to create a selective group using this criterion.

Finally, a negatively perceived atmosphere in class during the OVK program might have decreased the effectiveness of the program, since adolescents who perceive the atmosphere during the OVK lessons as negative, were more likely to have relatively higher levels of depressive symptoms. From our study is remains unclear what may have caused a negatively perceived atmosphere. In accordance with the expressed concerns of the teachers in the process evaluation, it might be that the content of the OVK program is less well covered when the atmosphere is negative, which may have reduced the impact of the program. Although, this effect could also be explained by a negative information bias that is hypothesized to be a vulnerability for developing depressive symptoms [49].

#### 3.5.2. Providers

Although the PRP program on which the OVK program is based was found to be effective when delivered by both teachers and professional interventionists [6], the current trial revealed no effects of our program when administered by teachers. Possibly, the teachers who administered our program reduced its effect, what would be in line with meta-analytic findings on several depression prevention programs, which showed that teachers are less effective in delivering prevention programs compared to professional interventionists [8], as is also found in a recent RCT which compared teachers and psychologist as providers [50]. One reason may be that teachers in our study received a four-day training in CBT and the specific OVK program techniques that might not have been sufficient to convey the curriculum’s content and the key teaching techniques in all its details, which could have decreased program fidelity. In some other depression prevention studies, teachers indeed received a more intensive training e.g., [44], and higher intervention fidelity could possibly have generated stronger effects [20]. On the other hand, other studies showed significant effects on depressive symptoms even though the training for the teachers in these programs was less intense [51]. However, even if the OVK program could be effective when teachers are trained more extensively, it may be unrealistic to expect teachers to invest in a longer training on top of their usual workload.

#### 3.5.3. Content

In the process evaluation of the OVK trial, teachers reported that the duration of the program (16 lessons) was too long. Specifically, the teachers reported difficulties in keeping the students motivated and emphasized that students complained that the lessons are boring. Additionally, teachers indicated to have too little time left for other classroom responsibilities. The adherence to the program, as reported by the teachers, was higher during the first half of the program compared to the second half of the program. However, we cannot draw strong conclusions on the effect of adherence level on program effectiveness, since not all teachers filled out adherence reports (12 of 28 did not). The skills based on CBT techniques might have been taught more thoroughly during the first half of the program compared to the second part, which focused on coping and social skills. It is possible that the coping and social skills are influential factors of the program and that their suboptimal implementation is in part responsible for the absence of the intervention effect. However, the content of CBT based programs was not found to influence the magnitude of the program effects in a meta-analytic review [8]. Still, based on our process evaluation and the finding that programs with a shorter duration tend to have larger effects [8], a shorter duration of the program might be more effective.

#### 3.5.4. Study Design

Although a review of CBT based depression prevention programs has not shown differences in the effect between study designs (e.g., amount of follow-up measurements) [8], the possible effect of adding long-term follow-up measurements after one year should be considered. We did not find an effect at one-year follow-up, although theoretically, it would be possible that a prevention effect would occur only after a longer period. Adolescents could then be confronted with situations in which they could use the learned skills and the adolescents or the control group might experience elevated depressive symptoms. However, in previous meta-analytic reviews, the strongest effect is found one year after the intervention [7,10]; therefore, the findings of our study might not have been different when using a longer follow-up period.

### 3.6. Moderation

Parental psychopathology moderated the relation between condition and clinical depressive symptoms. Adolescents who had parents with psychopathology and received the OVK program had less depressive symptoms compared to adolescents who had parents with psychopathology and were in the control condition. Although these findings are based only on 5.9% of the participants in the current study and thus a type I error could have occured, they add to previous research in which CBT interventions targeting adolescents with parents with psychopathology were found to be effective [52,53]. The selective group of adolescents with parents with psychopathology may be a particularly important subgroup for future prevention efforts.

### 3.7. Iatrogenic Effect

Besides the lack of a main effect of the OVK program on depressive symptoms, we found an iatrogenic effect on clinical depressive symptoms at one-year follow-up. We emphasize that this finding should be interpreted with caution because of the lack of robustness of this results when performing sensitivity analyses. Within the OVK group, we did not find differences in baseline characteristics between the adolescents who had clinical depressive symptoms at one-year follow-up and to those who did not. In addition, we neither found differences for motivation during the program and the perceived atmosphere. Accordingly, we can only speculate which adolescents might develop depressive symptoms more easily after following OVK. During the lessons, adolescents become more aware of their thoughts, feelings, and behavior, which may enhance their awareness of the presence of inappropriate depressive thoughts and feelings. This may in turn decrease their self-efficacy to deal with these thoughts and feelings and consequently increase depressive symptoms. Previous research has found that students low in general self-efficacy benefit more from a depression prevention program compared to their high self-efficacious peers [54]; however, whether a depression prevention program influences self-efficacy negatively and by consequence causes an iatrogenic effect would be of interest for further research.

### 3.8. Limitations

The present study should be interpreted in the light of its limitations. A first limitation involves the use of self-report questionnaires to measure depressive symptoms, which we did not compare with reports of parents or teachers. However, for internalizing disorders, such as depression, self-reports have been found to be more reliable [55]. Still, interviews are preferred in longitudinal research on depression [2] and research that aims to examine the effects of interventions on clinical diagnoses. Another limitation concerning the questionnaires is that parental psychopathology is measured by a single item in which the adolescent reports whether or not a parent is treated by a psychiatrist. Future research on the reliability of this measure is needed. Also a limitation of the study is that relatively more boys, minority youth, older pupils, and adolescents with higher depressive symptoms at baseline were lost to follow-up. Although most participants dropped out because they changed schools, the analysis indicated no difference in drop out between conditions and the analyses conducted separately for intention to treat and completers only did not reveal any differences, still, attrition might reduce the generalizability of our findings. Another limitation is that the study was non-blind; the teachers and adolescents knew whether they received the program or not. This could have resulted in a placebo effect of the program. However, a placebo effect is not likely to have occurred since we did not find an effect of the intervention. Moreover, adolescents were not notified about the goal of the study. Also, due to the nature of the study in which we did not apply an active control condition, it would be difficult keep the conditions unknown to the participants. Future studies are encouraged to apply a design using an active control condition and in which participants are blinded to the study conditions. In order to maximize the generalizability of the present study to the natural setting, we decided to keep the requirements for the teachers close to what would be a reasonable investment after a widespread intervention in real life. Therefore, we decided not to check teachers’ program integrity and adherence to the program, such as quality of delivery during the course of the study. Although we expect that audiotaping several lessons and checking the integrity [3,44] would increase the teachers’ efforts to deliver the program as mentioned, we expected that such checks will decrease schools and teachers’ willingness to adopt the program into their curriculum. Finally, with regard to the interaction effect of parental psychopathology, it is important to notice that only 5.9% of the participants reported their parents to have psychiatric problems. Because of possible power limitations this effect should be interpreted with caution and should be replicated in future studies.

## 4. Conclusions: Summary and Implications for Further Research

The OVK program administered by teachers in a school setting revealed no effects on depressive symptoms of adolescents from low-income areas in the Netherlands. Further research is recommeded, since previous research on depression prevention reported encouraging results. Because it can be questioned whether our population was actually a selective high-risk population, future selective prevention research should select participants that are more at risk than are adolescents from low-income areas, such as children of psychiatric parents or adolescents of an older age.
